# Visceral Leishmaniasis in a Non-endemic Region of Eritrea

**DOI:** 10.7759/cureus.11318

**Published:** 2020-11-03

**Authors:** Amin A Alamin

**Affiliations:** 1 Department of Pathology, College of Medicine, University of Al-Taif, Al-Taif, SAU

**Keywords:** visceral leishmaniasis, leishmania donovani, bone marrow aspiration, pancytopenia, asmara

## Abstract

Visceral leishmaniasis is endemic in the western states of Eritrea, but it is rare in the city of Asmara. We report a case of an 18-month-old female with a high-grade fever, weight loss, and hepatosplenomegaly. No obvious cause of her illness was found. Routine blood investigations showed pancytopenia, and microscopic examination of bone marrow revealed intracellular and extracellular Leishmania amastigotes, so a diagnosis of leishmaniasis (kala-azar) was finally made. Visceral leishmaniasis should be considered when a child presents with fever, weight loss, organomegaly, and pancytopenia.

## Introduction

No cases of visceral leishmaniasis were recorded before in Asmara city because it is not included in the endemic region of Eritrea. There is a lack of country-specific information in Eritrea. To the best of my knowledge, this article is the first article on visceral leishmaniasis in Asmara, Eritrea.

Visceral leishmaniasis (VL) is a parasitic disease caused by Leishmania donovani (L. donovani) infection after being bitten by a sandfly that has the parasite [1]. The disease affects the reticuloendothelial organs, specifically the bone marrow, liver, or spleen, and it can be fatal if misdiagnosed or not treated effectively. Its endemic areas in East Africa include Sudan, western states of Eritrea, Kenya, Ethiopia, Uganda, and Somalia [2]. Mohebali claimed that VL is characterized by anemia in extreme cases, loss of appetite, fever, liver and spleen enlargement, and loss of weight [3]. Depending on the condition, symptoms may or may not be visible in the early stage of the disease. The most common diagnostic method is microscopic confirmation of the presence of the parasite in the bone marrow [4]. Tissue smears of the spleen and lymph nodes can also show forms of L. donovani bodies (amastigote). However, splenic smears are more sensitive but painful, and riskier than lymph node and bone marrow smears [5]. Careful examination of the bone marrow helps identify intracellular and extracellular Leishmania bodies. This accurate and inexpensive diagnosis method is recommended when VL is suspected.

## Case presentation

An 18-month-old female from Asmara, Eritrea, had a two-month history of fever with sweating and chills, weight loss, progressive abdominal distention, and generalized mucocutaneous pallor. She did not respond to empirical antimalarial and antibiotics drugs and had no history of travel outside Asmara city. On examination, she had severe pallor, but there was no cyanosis or lymphadenopathy. Abdominal examination revealed mild hepatosplenomegaly, the spleen 4 cm below the coastal margin. The examination results of the other systems were unremarkable. The complete blood count results showed hemoglobin at 6.6 g/dL (normal: 10.5-13.5 g/dL), white blood cell count at 2.5 X 10^9/L (normal: 6.0-17.0 X 10^9/L), and platelet count at 80 X 10^3/L (normal: 150-450 X 10^3/L). The peripheral blood smear demonstrated pancytopenia and anisocytosis. The serological tests for malaria, tuberculosis, brucellosis, and salmonellosis were negative. 

**Figure 1:**
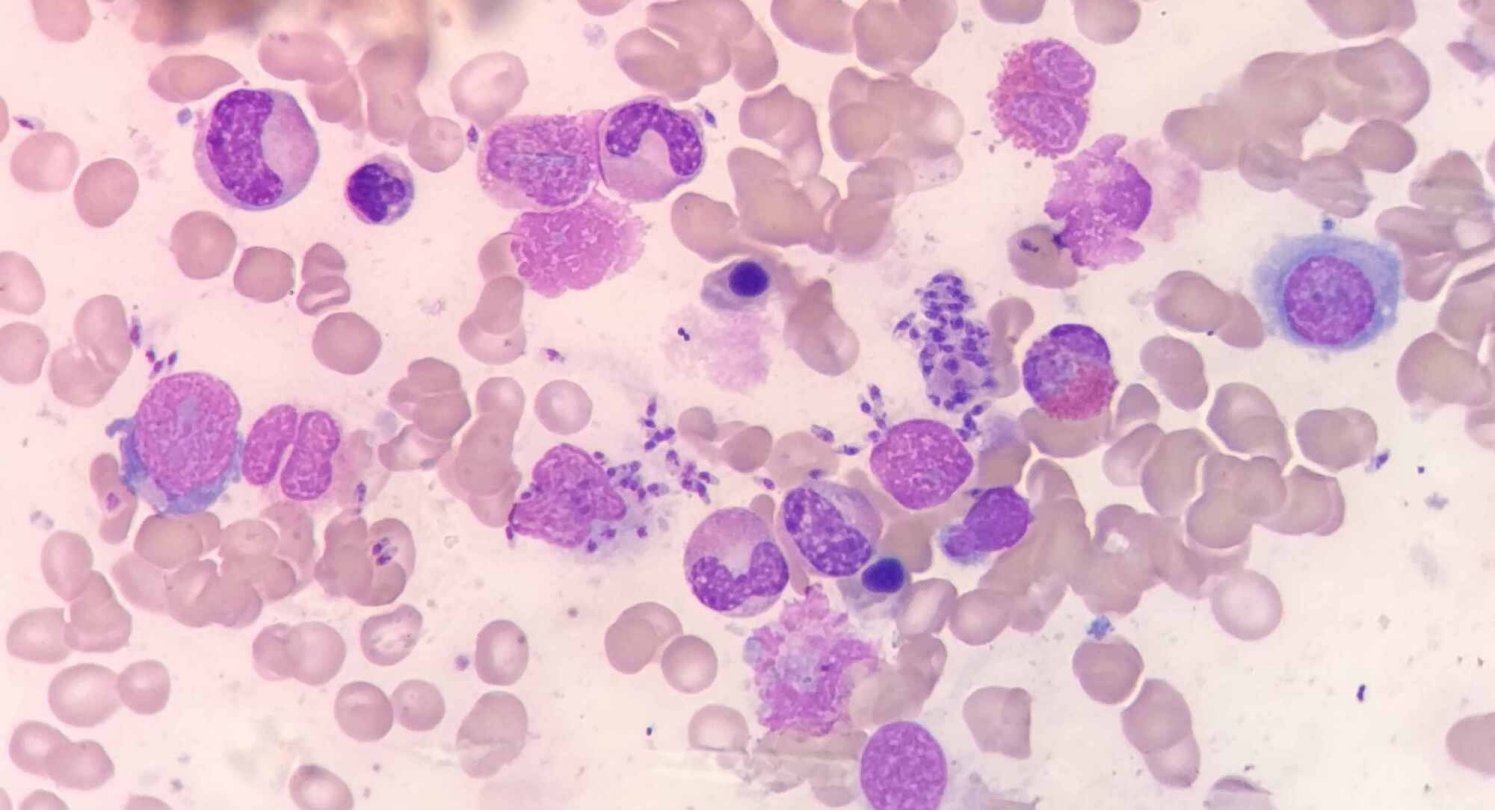
Leishmania donovani bodies intracellularly within the macrophages and extracellularly

**Figure 2:**
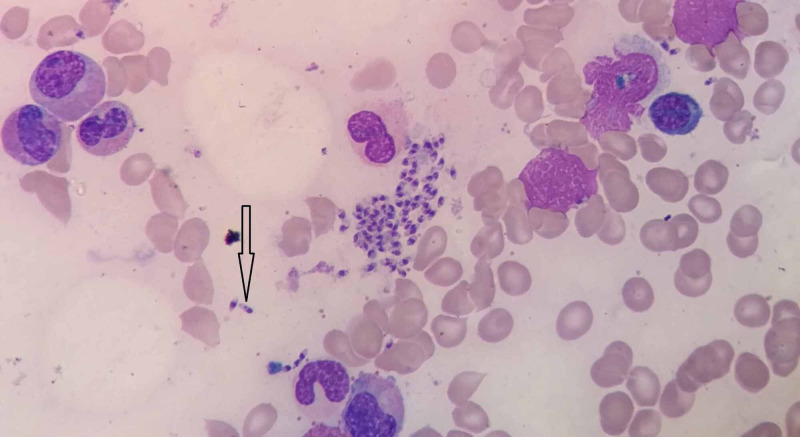
The Leishmania donovani bodies characterized by a kinetoplast and double dot appearance

Consequently, a bone marrow aspiration procedure was performed. Bone marrow smears revealed erythroid hyperplasia, with normoblastic erythropoiesis. The myeloid series was found to be active with moderate histiocytes and moderate dysplasia, and the plasma cell count was slightly increased. There was a prominent megakaryocytic series. There was an abundant amount of L. donovani bodies intracellularly within the macrophages and extracellularly (Figure 1). As seen in Figure 2, the L. donovani was characterized by a kinetoplast and double dot appearance. Based on the bone marrow examination results, the child was diagnosed as having VL (kala-azar) and immediately underwent treatment.

## Discussion

Leishmaniasis occurs in visceral and cutaneous forms, and the causative Leishmania species show a different geographical distribution depending on the environmental conditions. VL is caused by a parasite called Leishmania donovani [1]. Africa ranks second after India in terms of the number of cases of visceral leishmaniasis. Srivastava et al. argued that much of the disease in Africa is intense in the eastern part of that continent; L. donovani is endemic in the remote parts of Kenya, Uganda, Ethiopia, Somalia, and Sudan [6]. At least 4000 deaths are reported in this region each year. A total of 90% of the global cases of VL are found in Sudan, and there is also a high incidence of post-kala-azar dermal leishmaniasis in that country [2]. Acacia trees and termite hills, which are common in the region, provide breeding and resting places for certain species of sandflies. Proximity to them increases the risk of getting VL. The World Health Organization claimed that the Leishmania complex originated in Africa and it consists of L. donovani, L. tropica, L. aethiopica, L. infantum, and L. major [7].

In Eritrea, where the 18-month-old female child diagnosed with VL lives, there are limited control activities to decrease the spread of this disease [8]. Among the other ten countries in the region, Eritrea is included in the neglected tropical diseases (NTD) control activities created by the World Health Organization, which supports countries in lowering the transmission of VL. As noted by Al-Saleem, children are at a higher considerable risk of contracting visceral leishmaniasis in the region [9]. Because pastoralists and nomads spend a lot of time outside, they are more exposed to sandflies [2]. In the region, conditions, such as human immunodeficiency virus (HIV) and malnutrition, also increase the chances of contracting the disease. Diagnosis differs from one country to another, but microscopic examinations of bone marrow and spleen aspirates are most often used to confirm the presence of L. donovani bodies in the tissues [4]. Few treatment options are available because they are either expensive or challenging to administer [10]. As noted by Gebreyohannes, pentavalent antimonials and amphotericin B are some of the drugs that are commonly used in the region to treat VL [11]. There is a lack of knowledge on prevention measures, a lack of resources to intervene, and a lack of country-specific information in Eritrea.

## Conclusions

Visceral leishmaniasis should be considered when a child presents with fever, weight loss, organomegaly, and pancytopenia. Eritrea needs to support the control activities to decrease the spread of visceral leishmaniasis. Health education programs are required to improve knowledge on prevention measures.
